# A Facile Synthesis of 3-Substituted 9*H*-Pyrido[3,4-*b*]indol-1(2*H*)-one Derivatives from 3-Substituted β-Carbolines

**DOI:** 10.3390/molecules15085680

**Published:** 2010-08-17

**Authors:** Guowu Lin, Yue Wang, Qingfa Zhou, Weifang Tang, Jian Wang, Tao Lu

**Affiliations:** 1 College of Basic Science, China Pharmaceutical University, Nanjing 210009, China; 2 Key Laboratory of Drug Quality Control and Pharmacovigilance, Ministry of Education, China Pharmaceutical University, Nanjing 210009, China

**Keywords:** β-carboline, β-carbolinone, rearrangement, electron-withdrawing, X-ray crystal structure

## Abstract

A mild and efficient two-step synthesis of 3-substituted β-carbolinone derivatives from 3-substituted β-carboline in good yields is described. A possible reaction mechanism for the formation of the skeleton of β-carbolin-1-one is proposed. The structures of these compounds were established by IR, ^1^H-NMR, ^1^^3^C-NMR, mass spectrometry and elemental analysis, as well as X-ray crystallographic analysis of **4-2 **and **6-2**.

## Introduction

**Figure 1:**
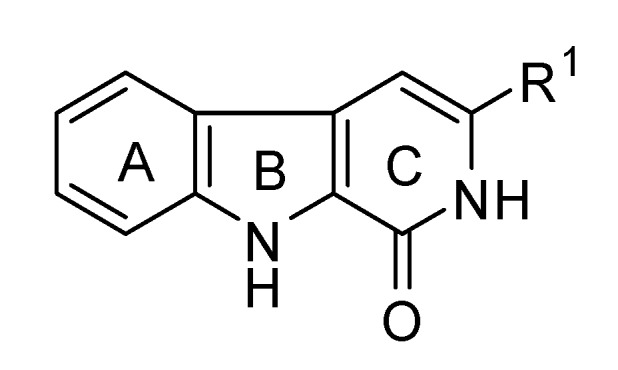
The structure of tricyclic β-carbolinones.

Natural products have a profound impact on both chemical biology and drug discovery, and the substituted β-carboline moiety is an example. β-Carboline is a key pharmacophore present in a large number of natural tricyclic alkaloids, which can be found in numerous plants and animals, exhibiting potent biological activities [1,2,3,4,5,6,7,8,9,10]. As a key member of the β-carboline family, its structural variant tricyclic β-carbolinone (9*H*-pyrido[3,4-*b*]indol-1(2*H*)-one derivative or β-carbolin-1-one, Figure 1) has served as an important intermediate for the preparation of complex alkaloids [11,12,13,14,15,16,17,18,19] and has been found to possess potent bioactivities. The natural and synthetic β-carbolinones are reported to have pharmacological effects in several aspects, such as the anticancer activity against colon and lung cancers, central nervous system activity in mammals, and also as the biological control agent for receptor research on bio-enzyme inhibitors, such as the inhibition of HLE (Human leukocyte elastase) [20,21,22,23]. In continuation of our work on biologically active β-carbolinone alkaloids [24], we focused our interest on the synthesis of 3-disubstituted β-carbolinones.

**Scheme 1:**
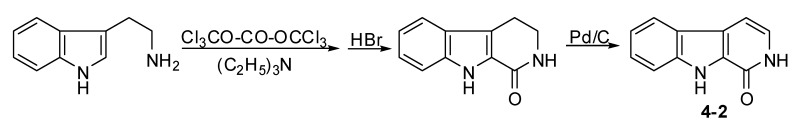
A conventional route to synthesize of 9*H*-pyrido[3,4-*b*]indol-1(2*H*)-one.

**Scheme 2:**
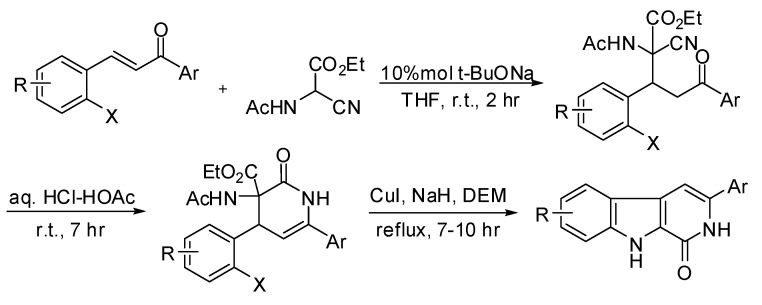
A route to synthesize 3-aryl-9*H*-pyrido[3,4-*b*]indol-1(2*H*)-one.

The total synthesis of these substituted tricyclic β-carbolinones has attracted great attention, however, few facile synthetic approaches to β-carbolinones have been published over the years. A majority of alkaloids are substituted at the 3-position of β-carbolinones, and two major synthetic strategies have been adopted for this purpose: one approach starting from tryptamine which offers an easy access to β-carbolinone (9*H*-pyrido[3,4-*b*]indol-1(2*H*)-one, Scheme 1) [25], and another three step route to synthesize 3-aryl-β-carbolinone by reaction of chalcone derivatives with *N*-acetyl-2-cyanoglycine ethyl ester (Scheme 2) [26]. Nevertheless, it is still difficult to introduce other groups directly at the 3-position of the β-carbolinone by these methodologies, especially for the synthesis of electron-withdrawing substituents at the 3-position of β-carbolinone. So it is urgent and significant to find a new and effective way to synthesize a large number of 3-substituted (electron-withdrawing) β-carbolinones. Herein, we report a novel synthetic route for the preparation of 3-substituted (electron-withdrawing substituents) β-carbolinones.

## Results and Discussion

**Scheme 3:**
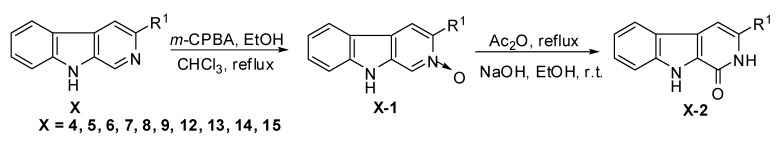
A route to synthesize 3-substituted β-carbolinones.

In this paper, we describe a two-step preparation of 3-substituted β-carbolinones using 3-substituted β-carbolines as the starting materials (Scheme 3). 

**Scheme 4:**
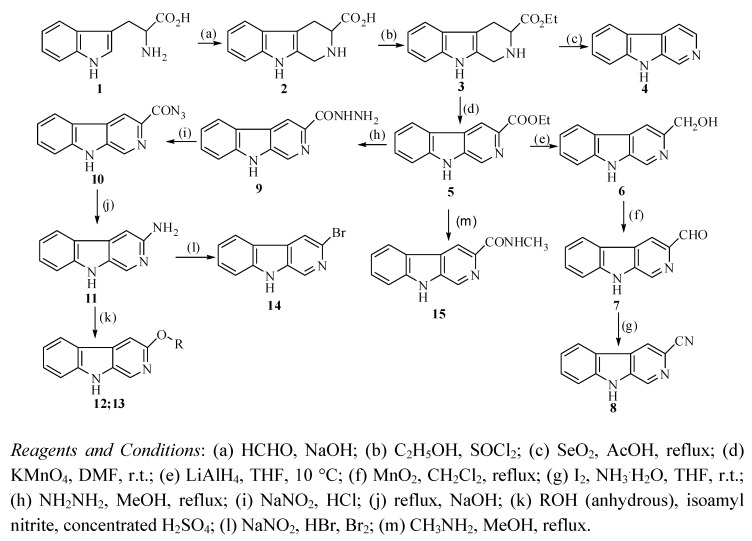
Synthesis of 3-substituted β-carbolines.

For the first step, in a mixed and refluxing 1:1 chloroform/ethanol solution, β-carboline derivatives **X** were treated with 3-chloro-peroxybenzoic acid (*m*-CPBA) yielding the corresponding *N*-oxides **X-1** in excellent yields, which then were purified by flash column chromatography; in the second step, the β-carbolinones were obtained through the regioselective rearrangement of the β-carboline-*N*-oxides in acetic anhydride at refluxing and subsequently hydrolysis in a solution of EtOH/aqueous 2 M NaOH (1:1) at room temperature. The 3-substituted β-carboline substrates were synthesized according to the literature procedure [27,28,29,30,31], which is summarized in Scheme 4.

**Scheme 5:**
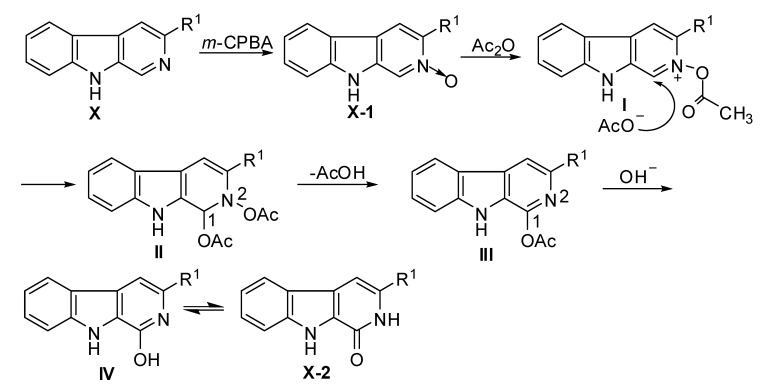
The possible mechanism for the synthesis of 3-substituted 9*H*-pyrido[3,4-*b*]indol-1(2*H*)-one derivatives.

Based on our experimental results and other similar reactions [32,33,34,35,36,37], the reaction mechanism of β-carboline *N*-oxides with glacial acetic acid and sodium hydroxide was presumed to be as shown in Scheme 5. The overall process may be summarized as follows: i) β-carboline-*N*-oxide **X-1** was refluxed with excess acetic anhydride to yield light brown syrup from which intermediate **I **(2-acetoxy-β-carboline derivative) was formed; ii) another acetyl oxygen anion (CH_3_COO^-^) attacks the carbon atom at 1-position of 2-acetoxy-β-carboline, and meanwhile the (C_1_ = N_2_) double bond was broken to give ntermediate **II **(1,2-diacetoxy-1,2-dihydro-β-carboline); iii) elimination of one molecule of acetic acid, and regeneration of the (C_1_ = N_2_) double bond to give intermediate **III **(1-acetoxy-β-carboline derivative)’ iv) by the hydrolysis of 1-acetoxy-β-carbolines **III** in sodium hydroxide solution, the target compounds were obtained via intermediate **IV.**

The various β-carbolines were used as substrates, and the results are summarized in Table 1. Under identical reaction conditions, using 3-ethoxycarbonyl-β-carboline, 3-hydroxymethyl-β-carboline, 3-cyano-β-carboline, β-carboline-3-carbohydrazide, or 3-(*N*-methylcarbamoyl)-β-carboline as starting materials, we obtained good yields (about 67-85%) of corresponding products (entries 2, 3, 5, 6 and 10 in Table 1). However, other β-carboline derivatives gave moderate yields (about 30-50%) of the corresponding β-carbolinones (entries 4, 7, 8 and 9). This discrepancy may be attributed to the different groups at the 3-position, because electron withdrawing substituents at the 3-position can enhance the electronic stability of intermediate **I**, and therefore, a relatively higher yield of product could be obtained. However, an electron-rich substituent is not beneficial for the electronic stability of intermediate **I**, so poor yield were observed in our experiments. All products were fully characterized by spectroscopic means.

**Table 1:** Yields and times for the synthesis of 9*H*-pyrido[3,4-*b*]indol-1(2*H*)-one derivative.

Entry	R^1^	Product (X-2)	Time (h)^ a^	Yield (%)^b,^^ c^
1	H	**(4-2)**	6	65
2	COOCH_2_CH_3_	**(5-2)**	4	85
3	CH_2_OH	**(6-2)**	8	75
4	CHO	**(7-2)**	6	52
5	CN	**(8-2)**	4	72
6	CONHNH_2_	**(9-2)**	4	67
7	OCH_3_	**(12-2)**	10	45
8	OCH_2_CH_3_	**(13-2)**	10	53
9	Br	**(14-2)**	12	30
10	CONHCH_3_	**(15-2)**	4	82

^a^ Monitored by TLC until *N*-oxidation is complete. ^b^ Isolated yield by column chromatography. ^c^ The yields of conversion of the *N*-oxides **X-1** into 3-substituted β-carbolinones **X-2**.

**Figure 2:**
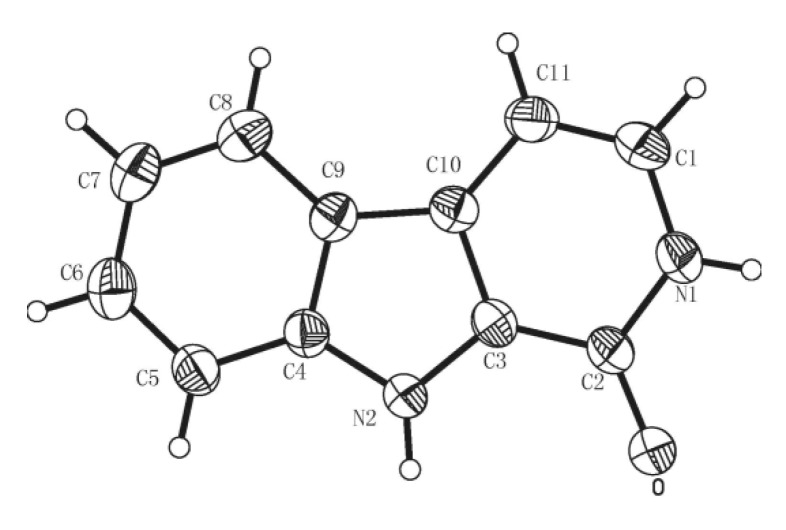
ORTEP drawing of the X-ray crystal structure of compound **4-2.** Displacement ellipsoids were drawn at 50% probability level.

**Figure 3:**
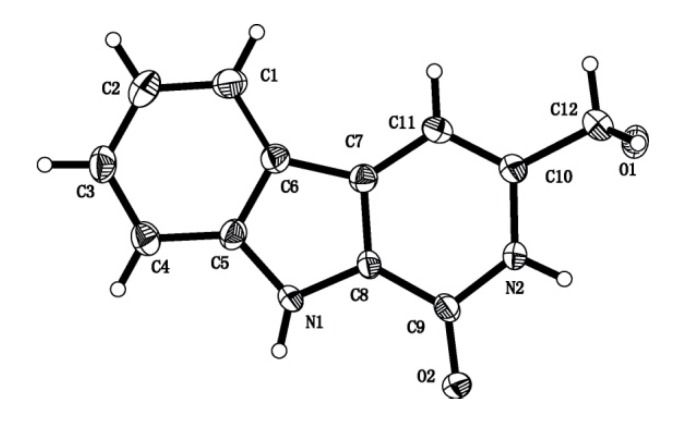
ORTEP drawing of the X-ray crystal structure of compound **6-2. **Displacement ellipsoids were drawn at 50% probability level.

At the same time, we obtained colorless platelet crystals of **4-2** from 80% (v/v) MeOH/H_2_O solution and **6-2** from 50% (v/v) MeOH/DMF (dimethylformamide) at room temperature (Figure 2-Figure 3) [38] for X-ray analysis.

## Conclusions

We have developed a simple and efficient two-step synthetic route for the synthesis in moderate to good yields of 3-substituted β-carbolinone derivatives from 3-substituted β-carbolines, and the reagents used are not hazardous and are easy to handle. This method offered a facile way to introduce various electron-withdrawing or electron-rich substituents into β-carbolinone derivatives at 3-position, which should broaden the application scope of the β-carbolinone skeleton. This type of reaction has been widely applied in our laboratory for the preparation of 3-substituted β-carbolinone derivatives. In addition, a plausible reaction mechanism has been proposed.

## Experimental

### General

Unless otherwise specified, reagents were purchased from commercial suppliers and used without further purification. Reaction progress was monitored using analytical thin layer chromatography (TLC) on percolated Merck silica gel Kiesegel 60 F254 plates, and the spots were detected under UV light (254 nm). Melting points were determined with a digital melting point apparatus and are reported uncorrected. ^1^H-NMR and ^13^C-NMR spectra were recorded at 300 MHz (^1^H) or 75 MHz (^13^C) on a Bruker ARX 300 spectrometer. IR spectra were measured on a Jasco FT/IR-430 spectrophotometer. Mass spectra were recorded on an a Quattro microMS Micromass UK mass spectrometer, and was recorded on an electrospray ionization mass spectrometer as the value m/z. The X-ray measurements were made on a Rigaku RAXIS RAPID diffractometer with a graphite monochromatised Mo Kα radiation (λ= 0.71069 Å) using ω scan mode. 

### General Procedure for the synthesis of N-oxidation

In a 100 mL single-necked, round-bottomed flask equipped with a magnetic stirrer, β-carboline derivative (1 mmol), 3-chloroperoxybenzoic acid (670 mg, 3 mmol), CHCl_3_ (5 mL) and EtOH (5 mL) were added. The reaction mixture was refluxed until there were no starting materials left (TLC monitoring), then cooled to room temperature. NaOH (3 mL, 0.1 M) were added and stirring was continued for 30 min. The aqueous phase was extracted with CHCl_3_ (2 × 25 mL), and the combined organic phases were dried with Mg_2_SO_4_ and concentrated in vacuo. The residues were purified by flash column chromatography using an eluent MeOH-CHCl_3_ to give the title compound as a pale yellow solid. Other compounds were synthesized similarly and the yields and spectroscopic data of **4-1**, **5-1**, **6-1**, **7-1**, **8-1**, **9-1, 12-1**, **13-1,** **14-1** and **15-1** were as follows.

*β-Carboline-N-oxide* (**4-1**). Yield: 175 mg (95%), pale yellow solid. ESI-MS m/z 185.10 (M + 1).

*3-Ethoxycarbonyl-β-carboline-N-oxide* (**5-1**). Yield: 238 mg (93%), pale yellow solid. ESI-MS m/z 257.45 (M + 1).

*3-Hydroxymethyl-β-carboline-N-oxide* (**6-1**). Yield: 190 mg (89%), pale yellow solid. ESI-MS m/z 215.20 (M + 1).

*3-Formyl-β-carboline-N-oxide* (**7-1**). Yield: 127 mg (60%), pale yellow solid. ESI-MS m/z 213.10 (M + 1).

*3-Cyano-β-carboline-N-oxide* (**8-1**). Yield: 188 mg (90%), pale yellow solid. ESI-MS m/z 210.05 (M + 1).

*β-Carboline-3-carbohydrazide-N-oxide* (**9-1**). Yield: 198 mg (82%), pale yellow solid. ESI-MS m/z 243.12 (M + 1).

*3-Methoxyl-β-carboline-N-oxide* (**12-1**). Yield: 160 mg (75%), pale yellow solid. ESI-MS m/z 215.05 (M + 1).

*3-Ethoxy-β-carboline-N-oxide* (**13-1**). Yield: 183 mg (80%), pale yellow solid. ESI-MS m/z 229.10 (M + 1).

*3-Bromo-β-carboline-N-oxide* (**14-1**). Yield: 174 mg (66%), pale yellow solid. ESI-MS m/z 264.15 (M + 1).

*3-(N-Methylcarbamoyl)-β-carboline-N-oxide*** (15-1**). Yield: 186 mg (87%), pale yellow solid. ESI-MS m/z 242.20 (M + 1)

### General Procedure for the synthesis of 9H-pyrido[3,4-b]indol-1(2H)-one derivatives

A β-carboline *N*-oxide derivative (1 mmol) were dissolved in acetic anhydride (10 mL), and the mixture was heated under reflux for 6 hours until there were no starting materials left (TLC control). After being cooled to ambient temperature, the reaction mixture was concentrated under reduced pressure. The intermediate 2-acetoxy-β-carboline derivative was dissolved in EtOH/aqueous 2 M NaOH (1:1), and stirred at room temperature for 2 hours. The solvent was removed *in vacuo*, and the residue was purified by column chromatography using MeOH/CHCl_3_ as an eluent to give the title pyridoindolone.

*9H-Pyrido[3,4-b]indol-1(2H)-one*** (4-2**): Prepared from **4-1**, yield 120 mg (65%), white solid, m.p. 253-255 °C. IR (KBr): 3261, 3112, 2966, 2840, 1645, 1446, 738 cm^-1^. ESI-MS m/z 185.05 (M + 1). ^1^H -NMR (DMSO-*d*_6_): δ = 11.91 (1H, s, indole), 11.35 (1H, s, -NHCO-), 8.03 (1H, d,* J *= 8.0 Hz, ArH), 7.50 (1H, d, *J *= 7.8 Hz, ArH), 7.39 (1H, t, *J *= 7.2 Hz, ArH), 7.17(1H, t, *J *= 8.0 Hz, ArH), 7.07 (1H, t, *J *= 7.3 Hz, ArH), 6.96 (1H, d, *J *= 7.0 Hz, ArH).^ 13^C-NMR (DMSO-*d*_6_): δ = 155.8, 139.1, 128.2, 126.3, 124.6, 124.4, 122.1, 121.4, 119.6, 112.6, 99.8. Anal. Calcd for C_11_H_8_N_2_O: C, 71.73; H, 4.38; N, 15.21. Found: C, 71.70; H, 4.37; N, 15.23.

*3-Ethoxycarbonyl-9H-pyrido[3,4-b]indol-1(2H)-one* (**5-2**): Prepared from **5-1**, yield 217 mg (85%), white solid. m.p. 257-259 °C. IR (KBr): 3380, 3128, 2985, 1701, 1652, 1298, 1249 cm^-1^. ESI-MS m/z 257.00 (M + 1). ^1^H -NMR (DMSO-*d*_6_): δ = 12.49 (1H, s, indole), 11.31 (1H, s, -NHCO-), 8.20 (1H, d, *J *= 8.3 Hz, ArH), 7.92 (1H, s, ArH), 7.55 (1H, d, *J *= 7.2 Hz, ArH), 7.46 (1H, t, *J *= 7.6 Hz, ArH), 7.26 (1H, t, *J *= 8.0 Hz, ArH), 4.30 (2H, q, *J *= 6.5 Hz,-OCH_2_), 1.30 (3H, t, *J *= 4.3 Hz, -CH_3_).^ 13^C-NMR (DMSO-*d*_6_): δ = 161.6, 154.8, 139.5, 131.2, 126.8, 125.0, 122.7, 122.2, 121.7, 120.7, 112.9, 106.2, 61.5, 14.3. Anal. Calcd for C_14_H_12_N_2_O_3_: C, 65.62; H, 4.72; N, 10.93. Found: C, 65.60; H, 4.75; N, 10.94

*3-Hydroxymethyl-9H-pyrido[3,4-b]indol-1(2H)-one* (**6-2**): Prepared from **6-1**, yield 160 mg (75%), white solid. m.p. 283-285 °C. IR (KBr): 3382, 3182, 3137, 2997, 1639, 1431, 1328, 744 cm^-1^; ESI-MS m/z 215.05 (M + 1). ^1^H-NMR (DMSO-*d*_6_): δ = 11.84 (1H, s, indole), 11.18 (1H, s, -NHCO-), 8.01 (1H, d, *J *= 7.9 Hz, ArH), 7.50 (1H, d, *J *= 7.2 Hz, ArH), 7.41 (1H, t, *J *= 8.0 Hz, ArH), 7.18 (1H, t, *J *= 7.3 Hz, ArH), 6.92 (1H, s, ArH), 5.30 (H, t, *J *= 6.1 Hz, -OH), 4.40 (2H, t, *J *= 3.5 Hz, -CH_2_O-).^ 13^C-NMR (DMSO-*d*_6_) : δ = 155.9, 139.4, 138.5, 126.9, 126.2, 124.4, 122.1, 121.3, 119.5, 112.6, 96.8, 60.3. Anal. Calcd for C_12_H_10_N_2_O_2_: C, 67.28; H, 4.71; N, 13.08. Found: C, 67.31; H, 4.65; N, 13.06.

*3-Formyl-9H-pyrido[3,4-b]indol-1(2H)-one* (**7-2**): Prepared from **7-1**, yield 110 mg (52%), white solid. m.p. 276-277 °C. IR (KBr): 3350, 3120, 2990, 1705, 1669, 1300 cm^-1^. ESI-MS m/z 213.10 (M + 1). ^1^H-NMR (300MHz, DMSO-*d*_6_): δ = 11.73 (1H, s, indole), 11.23 (1H, s, -NHCO-), 9.50 (2H, t, *J *= 7.8 Hz, -CHO), δ8.0 (1H, d, *J *= 8.0 Hz, ArH), 7.60 (1H, d, *J *= 7.2 Hz, ArH), 7.52 (1H, t, *J *= 7.0 Hz, ArH), 7.12 (1H, t, *J *= 8.1 Hz, ArH), 6.95 (1H, s, ArH).^ 13^C-NMR (300MHz, DMSO-*d*_6_): δ = 165.1, 152.1, 142.9, 140.1, 125.6, 124.6, 121.5, 120.6, 119.9, 119.3, 115.7, 114.2. Anal. Calcd for C_12_H_8_N_2_O_2_: C, 67.92; H, 3.80; N, 13.20. Found: C, 67.90; H, 3.83; N, 13.21.

*3-Cyano-9H-pyrido[3,4-b]indol-1(2H)-one* (**8-2**): Prepared from **8-1**, yield 150 mg (72%), white solid. m.p. 259-261 °C. IR (KBr): 3290, 3106, 2987, 2230, 1650, 1228 cm^-1^. ESI-MS m/z 210.30 (M + 1). ^1^H-NMR (DMSO-*d*_6_): δ = 12.05 (1H, s, indole), 11.31 (1H, s, -NHCO-), 8.11 (1H, d, *J *= 8.0 Hz, ArH), 7.69 (1H, d, *J *= 7.8 Hz, ArH), 7.45 (1H, t, *J *= 7.6 Hz, ArH), 7.20 (1H, t, *J *= 8.2 Hz, ArH), 6.93 (1H, s, ArH).^ 13^C-NMR (DMSO-*d*_6_): δ = 155.3, 145.2, 138.1, 126.9, 125.8, 124.4, 122.9, 121.6, 119.6, 119.0, 115.7, 115.2. Anal. Calcd for C_12_H_7_N_3_O: C, 68.89; H, 3.37; N, 20.09. Found: C, 68.90; H, 3.33; N, 20.11.

*9H-Pyrido[3,4-b]indol-1(2H)-one-3-carbohydrazide* (**9-2**): Prepared from **9-1**, yield 190 mg (67%), white solid. m.p. 303-305 °C. IR (KBr): 3386, 3251, 3053, 2979, 2935, 1718, 1629, 1245, 738 cm^-1^. ESI-MS m/z 243.30 (M + 1). ^1^H-NMR (DMSO-*d*_6_): δ = 12.13 (1H, s, indole), 11.70 (1H, s, -NHCO-), 9.01(1H, s, -NH), 8.05 (1H, d, *J *= 7.9 Hz, ArH), 7.45 (1H, d, *J *= 7.0 Hz, ArH), 7.39 (1H, t, *J *= 8.0 Hz, ArH), 7.10 (1H, t, *J *= 7.9 Hz, ArH), 6.90 (1H, s, ArH), 4.50(2H, s, -NH_2_).^ 13^C-NMR (DMSO-*d*_6_): δ = 167.2, 153.2, 142.8, 139.5, 126.5, 122.4, 121.9, 121.3, 119.9, 119.6, 114.7, 111.4. Anal. Calcd for C_12_H_10_N_4_O_2_: C, 59.50; H, 4.16; N, 23.13. Found: C, 59.48; H, 4.19; N, 23.12.

*3-Methoxy-9H-pyrido[3,4-b]indol-1(2H)-one* (**12-2**): Prepared from **12-1**, yield 96 mg (45%), white solid. m.p. 180-181 °C. IR (KBr): 3360, 3120, 2860, 1665, 1290, 1050 cm^-1^. ESI-MS m/z 215.00 (M + 1). ^1^H-NMR (DMSO-*d*_6_): δ = 11.62 (1H, s, indole), 11.50 (1H, s, -NHCO-), 7.98 (1H, d, *J *= 7.8 Hz, ArH), 7.42 (1H, d, *J *= 8.1 Hz, ArH), 7.35 (1H, t, *J *= 7.2 Hz, ArH), 7.10 (1H, t, *J *= 8.1 Hz, ArH), 6.33 (1H, s, ArH), 3.80 (3H, s, -OCH_3_).^ 13^C-NMR (DMSO-*d*_6_): δ = 156.5, 152.0, 142.1, 139.5, 124.5, 122.4, 121.9, 120.6, 119.6, 114.7, 104.1, 60.5. Anal. Calcd for C_12_H_10_N_2_O_2_: C, 67.28; H, 4.71; N, 13.08. Found: C, 67.30; H, 4.70; N, 13.07.

*3-Ethoxy-9H-pyrido[3,4-b]indol-1(2H)-one* (**13-2**): Prepared from **13-1**, yield 120 mg (53%), white solid. m.p. 183-184 °C. IR (KBr): 3365, 3250, 2900, 1640, 1300, 1100 cm^-1^. ESI-MS m/z 229.00 (M + 1). ^1^H-NMR (DMSO-*d*_6_): δ = 11.71 (1H, s, indole), 11.43 (1H, s, -NHCO-), 8.05 (1H, d, *J *= 8.0 Hz, ArH), 7.45 (1H, d, *J *= 7.3 Hz, ArH), 7.37 (1H, t, *J *= 7.9 Hz, ArH), 7.10 (1H, t, *J *= 8.0 Hz, ArH), 6.55 (1H, s, ArH), 4.0 (2H, q, *J *= 4.6 Hz, -OCH_2_) , 1.3 (3H, t, *J *= 5.1 Hz, -CH_3_).^ 13^C-NMR (DMSO-*d*_6_): δ = 156.7, 152.2, 142.5, 139.3, 125.0, 122.3, 121.7, 120.8, 119.6, 115.3, 103.6, 69.0, 19.7. Anal. Calcd for C_13_H_12_N_2_O_2_: C, 68.41; H, 5.30; N, 12.27. Found: C, 68.20; H, 5.55; N, 12.23.

*3-Bromo-9H-pyrido[3,4-b]indol-1(2H)-one* (**14-2**): Prepared from 1 **4-1**, yield 80 mg (30%), pale yellow solid. m.p. 270-271 °C. IR (KBr): 3382, 3207, 2921, 1660, 1299 cm^-1^; ESI-MS m/z 264.50 (M + 1). ^1^H-NMR (DMSO-*d*_6_): δ = 12.13 (1H, s, indole), 11.45 (1H, s, -NHCO-), 8.00 (1H, d, *J *= 7.7 Hz, ArH), 7.54 (1H, d, *J *= 8.0 Hz, ArH), 7.41 (1H, t, *J *= 8.7 Hz, ArH), 7.05 (1H, t, *J *= 7.2 Hz, ArH), 6.67 (1H, s, ArH). ^13^C-NMR (DMSO-*d*_6_): δ = 155.7, 141.9, 139.6, 126.3, 122.0, 121.8 120.4, 119.7, 116.7, 114.5, 109.3. Anal. Calcd for C_11_H_7_BrN_2_O: C, 50.22; H, 2.68; N, 10.65. Found: C, 50.20; H, 2.69; N, 10.67.

*3-(N-Methylcarbamoyl)-9H-pyrido[3,4-b]indol-1(2H)-one* (**15-2**): Prepared from **15-1**, yield 197 mg (82%), white solid. m.p. 282-283 °C. IR (KBr) 3360, 3320, 3280, 2990, 1710, 1660, 1314 cm^-1^; ESI-MS m/z 242.20 (M + 1). ^1^H-NMR (DMSO-*d_6_*): δ = 11.70 (1H, s, indole), 11.25 (1H, s, -NHCO-), 8.88 (1H, s, -CONHCH_3_), 8.05 (1H, d, *J *= 8.0 Hz, ArH), 7.55 (1H, d, *J *= 7.9 Hz, ArH), 7.45 (1H, t, *J *= 7.2 Hz, ArH), 7.20 (1H, t, *J *= 7.6 Hz, ArH), 6.85 (1H, s, ArH), 2.80 (3H, d, *J *= 4.3 Hz, -CH_3_).^ 13^C-NMR (DMSO-*d*_6_): δ = 162.7, 155.3, 141.9, 127.7, 123.2, 121.8, 121.5,121.1, 119.9, 118.8, 114.3, 112.7, 36.2. Anal. Calcd for C_13_H_11_N_3_O_2_: C, 64.72; H, 4.60; N, 17.42. Found: C, C, 64.75; H, 4.58; N, 17.40.
